# Mentorship for research on TB in India: a course on Public Health Implementation Strengthening and Operational Research (MPHISOR)

**DOI:** 10.5588/pha.25.0059

**Published:** 2026-08-24

**Authors:** H.D. Shewade, P. Manickam, T. Bhatnagar, K. Shringarpure, M. Das, S. Kalaiselvi, D. Nair, J.M. Melfha, M. Verma, K.M. Akshaya, S.K. Mattoo, M.V. Murhekar

**Affiliations:** 1 ICMR National Institute of Epidemiology (ICMR-NIE), Chennai, India;; 2 Medical College Baroda, Vadodara, India;; 3 FIND, New Delhi, India;; 4 All India Institute of Medical Sciences (AIIMS), Madurai, India;; 5 ICMR National Institute for Research in Tuberculosis (ICMR-NIRT), Chennai, India;; 6 All India Institute of Medical Sciences (AIIMS), Bathinda, India;; 7 Yenepoya Medical College, Mangaluru, India;; 8 Central TB Division, New Delhi, India.

**Keywords:** tuberculosis, capacity building, operational research, SORT IT, MPHISOR

## Abstract

This issue of *Public Health Action* includes a series of articles on operational research (OR) on various aspects of ‘TB in India’. These are the outputs of a two-year mentored course. Year one was based on the Structured Operational Research and Training Initiative (SORT IT), a WHO/TDR-accredited model for building in-country OR capacity. Building on this work, year two was an innovation implemented by ICMR National Institute of Epidemiology (ICMR-NIE), in which mentees implemented a second mentored OR project. In this way, they did not just identify a problem but attempted to address it, to increase the chances of achieving even greater policy/practice change.

Operational research (OR) generates practical usable knowledge which improves health program implementation, regardless of the type of research. The Structured Operational Research and Training IniTiative (SORT IT) is a WHO/TDR-accredited model for building in-country OR capacity.^1^ It is an output-based, one-year course where mentees are competitively selected, complete three weeks of residential modular training, and conduct a mentored OR project (to be completed within one-year period) on a program-relevant OR question (bridging the know-do gap). A SORT IT course can be defined by a geographic region, by the topic/disease condition/health program and number of mentees (usually 12). The three modules (one week each) are on protocol development (module one), data capture tools and analysis methods (module two) and manuscript writing (module three).^1^ Recently, a fourth module on effective research communication for decision makers has been introduced. After submitting their manuscript to a peer-reviewed scientific journal, mentees successfully complete the course and are eligible for the SORT IT certificate.^1^ To implement a SORT IT course it must meet the 80-80-80-80 targets: i) average mentee score for each module should be at least 80%, ii) at least 80% of the mentees should successfully complete the course, iii) 80% of submitted manuscripts should be published within 18 months and iv) 80% of submitted manuscripts should be assessed for policy/practice change (self-reported by mentees with means of verification).^1^

In the past, WHO/TDR attempted to document policy/practice changes by conducting a follow up SORT IT course, where a team member associated with a previous SORT IT underwent a follow up course. As an ICMR National Institute of Epidemiology (ICMR-NIE) innovation, we have developed this approach and established a two-year course. Mentees built on their work in year one (2024–2025) and conducted a second mentored OR project in year two (2025–2026). Successful completion of year one made the mentees eligible for a SORT IT certificate and allowed them to enter year two. Successful completion of year two made them eligible for a Mentorship for Public Health Implementation and Operational Research (MPHISOR) certificate. In the second year, the mentees could i) conduct follow-up OR guided by the year one findings, ii) document changes following recommendations from year one, or iii) answer another but related OR question. The two-year duration was conceptualized from the start in the call for applications, giving an opportunity for the applicants to think long-term and propose two OR questions.

We received approximately 80 applications of which 12 were competitively selected. In year one, the mentees implemented 12 mentored OR projects, and all successfully completed SORT IT and submitted manuscripts (one mentee submitted 2 manuscripts), of which 12 were published (Table). Unfortunately, one mentee was unable to continue (due to an internal transfer in the state), and in year two, 11 mentees implemented 11 mentored OR projects, and 9 successfully completed MPHISOR. For the SORT IT rating we achieved 92-108-100 and, using the same SORT IT targets for MPHISOR, we achieved 93-83-100 (based on 11 students attending the course).

**TABLE.** Outputs of ICMR-NIE TB SORT IT course (2024–2026).

	SORT IT 2024–2025	MPHISOR 2025–2026
Mentees	12	11**^A^**
Mentored projects	12	11
Manuscripts submitted	13	9
Published	12	9

A Of 12 mentees in year one (2024–2025), 11 continued into year two (2025-2026).

ICMR = Indian Council of Medical Research; NIE: National Institute of Epidemiology; SORT IT = Structured Operational Research and Training IniTiative (WHO/TDR accredited); MPHISOR = Mentorship for Public Health Implementation Strengthening and Operational Research (ICMR-NIE innovation).

This two-year model demanded sustained commitment from mentees, mentors and program compared to the traditional one-year SORT IT model. Two years increased the chances for meaningful engagement with program leading to policy/practice change. We did not just end by identifying a problem, but tried to address it. A senior officer from the national TB elimination program coordinated the dissemination of the call for applications and communicated with the states after the year two OR projects were finalized. We introduced mentor site visits in the second year to advocate for local action. We did not implement module two in the second year and module four in both the years. Though there was no module four, all mentees developed one-page lay person summaries and lightning presentation slides. Half of the mentees recorded lightning presentation videos. In addition to in-person technical presentations, this advocacy material was also shared with state level program managers.

## TB IN INDIA

Of the 9 MPHISOR manuscripts published in this issue of the Journal, 8 were follow-up OR guided by the findings of year one,^2-9^ and 1 answered another related OR question.^10^ Abdullah et al. initially documented the potential of digital annual household survey data to prioritize high-risk populations for active case-finding in Rajasthan.^2^ This was followed by data-driven active case-finding for TB in a district.^11^ Gupta et al. observed that district level private cough syrup sales correlated positively with missed TB notification in the private sector in Rajasthan.^3^ This guided a private pharmacy-based TB surveillance pilot in a district with high cough syrup sales.^12^ Guided by their statewide OR in Chhattisgarh,^4^ Malik et al. implemented statewide decentralized household contact investigation along with the inclusion of monitoring indicators in quarterly performance reviews.^13^ Based on findings of high burden and inequity in coverage of existing nutrition supplementation schemes among adults with TB with severe and very severe undernutrition in Chhattisgarh,^5^ Gupta et al. successfully advocated for a state level TB-undernutrition guidance.^14^ Patnaik et al. identified major electronic data capture issues in documenting TB screening among people living with HIV in Odisha.^6^ In year two, the reasons behind this were explored among the end-users.^15^ Jeyakumar et al. implemented a quality improvement package following the findings of suboptimal triage quality (for severe illness) at diagnosis in some districts of Tamil Nadu that did not reduce TB deaths following a statewide differentiated TB care initiative to reduce TB deaths.^7,16^ Natarajan et al. applied the findings of first year OR to guide a pilot on screening for anti-TB drug induced liver injury among high-risk groups in four districts of Tamil Nadu.^8,17^ Gouroumourty et al. implemented an initiative to address inequities in food basket distribution in Puducherry.^9,18^ Gupta et al. assessed the effect of a patient provider support agency model on notification and service provision in the private sector in India using two different designs, one each in each year of the course.^10,19^

With the support of mentors and the national TB program, ICMR-NIE successfully conducted the two-year national OR capacity building course on TB in India. All 12 mentees successfully completed the SORT IT course and 9 completed the MPHISOR course. In total, there were 23 mentored OR projects and 21 OR manuscripts published (see Table). It is important to note that all the projects were conducted in routine program settings using existing resources and workforce. However, these are early implementation findings, and long-term follow up is required to assess whether the changes are sustained over time.

## CONCLUSION

Subject to commitment by the health program and technical support from a research/academic institute experienced in OR capacity building (i.e., one-year SORT IT courses), we believe the year two MPHISOR course could be replicated elsewhere. This second year of study increased the chances of meaningful engagement with the program in the hope of achieving even greater policy/practice change.
